# Application of the Chinese version of Zelaya’s HIV-related stigma scale to undergraduates in mainland China

**DOI:** 10.1186/s12889-019-8054-9

**Published:** 2019-12-19

**Authors:** Fang Ruan, Guochen Fu, Mingyu Zhou, Lan Luo, Jing Chen, Wei Hua, Xin Li, Yifan Chen, Xiaobao Xia, Yanting Xiong, Yuhua Chen, Bin Shi, Shengbo Lu, Hudie Zhang, Dawei Wu, Yusi Liu, Jihong Zhan, Junfang Wang

**Affiliations:** 1 Department of Preventive Medicine, School of Basic Medical Sciences, Hubei University of Science and Technology, No.88 Xianning Avenue, Xianning City, 437100 Hubei Province China; 2 National Demonstration Center for Experimental General Medicine Education of Hubei University of Science and Technology, No.88 Xianning Avenue, Xianning City, 437100 Hubei Province China

**Keywords:** HIV-related stigma, Internal consistency, Construct validity, Exploratory factor analyses, Confirmatory factor analyses

## Abstract

**Background:**

This cross-sectional study aims to validate the Chinese version of Zelaya’s HIV-related Stigma Scale (CVZHSS) among a large undergraduate sample in mainland China, and apply it to measure the level of different dimensions of stigma and their respective determinants.

**Methods:**

From September 10, 2018, to January 9, 2019, a total of 10,665 eligible undergraduates conveniently drawn from 30 provinces in mainland China (except for Tibet) completed the self-designed online questionnaire distributed via sojump.com voluntarily, anonymously and confidentially. Both exploratory and confirmatory factor analyses (EFA and CFA) were first performed to test its construct validity, Cronbach’s alpha was then used to assess its internal consistency, and Logistic regression analyses were finally carried out to identify predictors of various dimensions of stigma.

**Results:**

As expected from the original model, four factors (i.e., “fear of casual transmission”, “moral judgment”, “personal stigma” and “perceived community stigma”) were extracted using principal component analysis with varimax rotation, accounting for 63.26% of the total variance. The CFA further confirmed the four-factor construct (CFI = 0.92, GFI = 0.91, RMSEA = 0.07). In addition, all the four factors demonstrated acceptable internal consistency with Cronbach’s alpha ranging from 0.83 to 0.92. Stigma as measured by “fear of casual transmission” (74.4%), “moral judgement” (61.6%), “personal stigma” (79.0%) and “perceived community stigma”(36.5%) is highly prevalent among undergraduates. Except for non-freshmen, less knowledge about HIV and unsafe sex which were consistently associated with higher levels of stigma in all four dimensions, other eight variables including gender, residential area, major, sexual orientation, having ever being tested perception of HIV risk, willingness to utilize HTC service and awareness of the national AIDS policy played differential roles in affecting different dimensions of stigma.

**Conclusions:**

The CVZHSS is a reliable and valid measurement tool and can be used to identify undergraduates with high levels of stigma. However, the four dimensions (Fear, moral judgement, personal stigma and perceived community stigma) were respectively influenced by different determinants, and thus should be treated independently when designing, implementing and evaluating stigma reduction programs.

## Background

For nearly 40 years since the first case was identified in 1980s, stigma against people living with HIV (PLWH) has been documented as a major barrier to HIV- related prevention, diagnosis, care and treatment [1], which not only inhibits high-risk individuals from reducing their risk behaviors, getting tested for the virus and disclosing their positive status, but also causes delays in their care-seeking behavior, diagnosis and treatment initiation [2, 3]. Elimination of the stigma is critical to achieve the “90–90-90” targets by 2020 and finally to end the AIDS epidemic by 2030 [4].

Recently, college students in China have been chosen as a key target population for anti-stigma and anti-discrimination intervention, because they are among the most vulnerable to HIV infection due to their earlier initiation of sexual activity and subsequent unsafe sexual behaviors such as having multiple sex partners, having sex with casual or commercial sex partners, and inconsistent condom use [5–8]. For example, a cross -sectional study based on a national large sample of 18,000 Chinese college students [5] and our most recently published study [6] have demonstrated that nearly one-third of sexually active college students started their sexual activity earlier than 18 years of age. Furthermore, early sexual initiators, whether male [7] or female [8], were found to have a higher likelihood than late initiators to have sex with multiple sexual partners or non-regular partners, and fail to use condom consistently.

Various paradigms, approaches and techniques have been attempted to define, conceptualize, measure and provide solutions to HIV-related stigma, and has also yielded valuable results [9–16], including the design and development of a great number of scales and accompanying questions to measure and compare different dimensions of stigma in a variety of cultural contexts and with the three most commonly-mentioned groups (i..e, healthcare workers, HIV-infected individuals and the general population). However, the lack of a standardized instrument makes it difficult to measure stigma consistently and thus poses a challenge to compare and contrast evaluated interventions [4, 17, 18]. Furthermore, although scales were adapted to different linguistic and sociocultural contexts, their reliability and validity were rarely tested [12]. Therefore, there is a need for developing a standardized, reliable and valid measurement tool to assess the current level and predictors of HIV-related stigma, and subsequently develop, implement and evaluate anti-stigma interventions.

A review of the existing literature indicated that there existed a multitude of candidate HIV-related scale items that can be used or adapted for use in the undergraduate student population in China [19–29]. However, only a few scales were provided with the evidence of validity and reliability [21–23, 26, 27], and none of these scales had been assessed in a large, nationally diverse sample of undergraduates. For instance, the discrimination related HIV/AIDS scale [22], which was developed by Cao and her colleagues in 2013 and consisted of 19 items with four dimensions (Fear, avoidance, disclosure and moral judgment), had ever been tested by using only exploratory factor analysis in a sample of 449 medical and nursing students from Nantong University. Similarly, the 15-item scale designed by Yang, Wang and Yuan [26] to measure three dimensions of stigma (Fear, moral judgment, personal stigma) and the 29-item scale covering the three dimensions of stigma (social distance, moral judgment, and personal stigma) designed by Qian and Wang [27] were also verified in a small sample of college students, although further evidence of construct validity was provided by both exploratory and confirmatory factor analyses. In comparison, the Chinese version of Zelaya’s HIV-related Stigma Scale (CVZHSS), which was translated by Xing and colleagues in 2012 [23] and yielded a four-factor structure and successfully measured the four dimensions of stigma [including fear of casual transmission, moral judgment such as shame and blame, personal stigma (i.e., personal beliefs and feelings related to fair treatment of PLWH in a society), and perceived community stigma (i.e., the respondents’ perceptions of how people in a community feel and respond towards PLWH)] as expected from the original scale by using traditional exploratory factor analysis, seems to be a promising measurement tool because it has some advantages over the above-mentioned three scales in several important ways. First, it assessed stigma more comprehensively, because it measured “perceived community stigma” reported by HIV-uninfected individuals, besides highlighting the three most commonly included dimensions (Fear, moral judgment and personal stigma) [30]. Second, the CVZHSS has been employed globally to assess HIV stigma among individuals who do not know their HIV status {including but not limited to college students [21, 23, 24] and rural-urban migrants in China [31], correctional staff in prisons and jails [32] and adults and adolescents [33] in the United States, male bar patrons [30] and nursing students [34] in India} for more than 10 years due to its flexibility to adapt to varying linguistic and sociocultural contexts and also because of its diversity in the measurement of its four dimensions. Third, it has been well-validated in a relatively large sample of medical college students in Zhejiang province of China [23] and used fairly extensively in Chinese college students majoring in nursing [21], medicine [23, 24] and education [24].

**Fig. 1:**
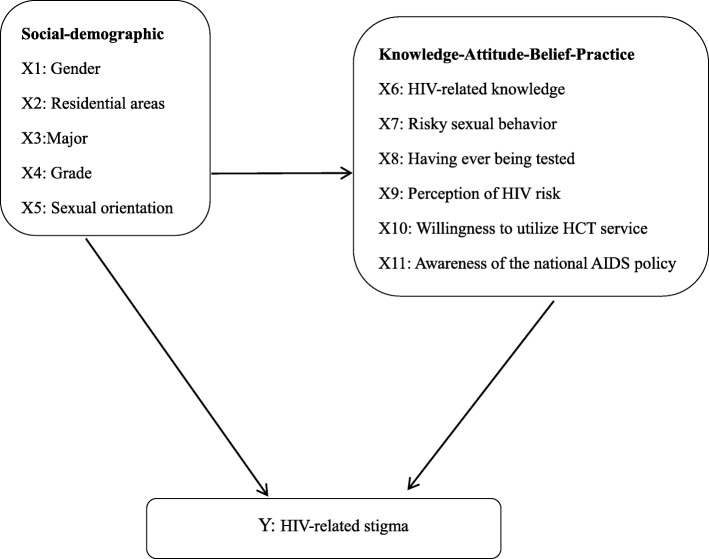
Individual determinants of HIV-related stigma

While the CVZHSS has been tested and validated successfully for medical college students in Zhejiang province of China and also applied to undergraduates majoring in nursing, medicine and education, its reliability and validity among general college students (including but not limited to college students majoring in medicine, nursing and education) throughout mainland China had not been fully tested prior to the current research. Moreover, eleven factors (See Fig. 1), including five social-demographic characteristics {gender [19], residential areas [19], major [24], age [35], and sexual orientation [35]}, HIV-related knowledge [19], awareness of the national AIDS policy [36], risky sexual behaviors [37], three preventive practices [37] such as having ever being tested for HIV [38], willingness to utilize HIV testing and counselling (HTC) service [37] and perception of HIV risk [36], have previously been studied as correlates of HIV-related stigma. However, these studies usually used bivariate analyses that did not take into account the effects of potential confounders. Thus, independent effects of eleven variables on stigma remain unclear. The present study aims to first validate the Chinese version of Zelaya’s HIV-related Stigma Scale (CVZHSS) among a large undergraduate sample in mainland China, and then apply it to measure the level of different dimensions of stigma and their respective determinants. There are two main goals of this article: (1) to better understand the mechanisms of how stigma is processed, and (2) to better understand the development, implementation and evaluation of anti-stigma interventions to achieve the ambitious goal of “Zero Discrimination” [39].

## Methods

### Data collection

Due to its convenience, cheapness and flexibility in both time and space, an online survey was carried out to collect the data about HIV-related stigma and associated factors among undergraduates in mainland China. However, some disadvantages such as multiple completions by the same individual and ineligible respondents entering the sample cannot be ignored [40]. Therefore, we prevented duplicate participation from the same person by limiting access from the same IP address and the present analysis was restricted to undergraduates in mainland China, who must meet the following four inclusion criteria: (a) answered the questionnaire no later than January 9, 2019; (b) age 18–25 years; (c) currently registered at one university in mainland China; (d) full-time undergraduates.

This protocol was approved by the academic ethics and moral supervision committee from Hubei University of Science and Technology (HUSC). The data collecting method has been already described in our previous paper [6]. Briefly, both convenience sampling and snowball sampling techniques were applied to select the participants. Firstly, undergraduates from HUST were conveniently recruited to complete the online questionnaire distributed via the website “https://www.wjx.cn/“. Meanwhile, existing undergraduates were encouraged to recruit future undergraduates from among their acquaintances to participate in the online survey for credits and even earning the honor of “outstanding volunteers”. Furthermore, Wechat, Sina Weibo, and QQ space were also chosen as the platforms to distribute the survey link in order to obtain a more geographically diverse sample. After signing electronic informed consent voluntarily, participants completed the anonymous questionnaire and were also promised that all the information they provided would be treated confidentially and only used for academic research.

### Measures

#### Dependent variable-HIV-related stigma

The dependent variable of interest (HIV-related stigma) in this study was measured with the Chinese version of Zelaya’s HIV-related stigma scale (CVZHSS). This scale [23, 30] is composed of four domains, each with six items (Table 2). The “Fear of casual transmission” domain (Items1–6) reflected people’s fear of being infected through casual contact with a person living with HIV (PLWH) such as kissing the cheeks, being exposed to cough, sneeze, saliva, sweat and urine, or playing with an HIV-infected person. The “moral judgment” domain (Items7–12) intends to capture shame and blame associated with HIV and behavior considered to be immorally by social standard (e.g., PLWH should be blamed, punished, condemned or held responsible for being HIV-positive). The “Personal stigma” domain (Items13–18) measured their personal beliefs and feelings related to fair treatment of PLWH in a society (e.g., PLWH should be legally separated from others to protect the public health), while the “Perceived community stigma” domain (Items19–24) reflected the respondents’ perceptions of how people in a community feel and respond towards PLWH (e.g., PLWH should be abandoned by his his spouse or partner).

Consistent with our previous study [36], participants were asked to indicate the extent to which they expressed their fears/agreement when facing the above- mentioned 24 situations and three alternative responses (Yes, No, It depends on the situation) were provided for each question. For all items above, “It depends on the situation” responses were scored as incorrect with a zero, while correct responses were scored as one (all items were reversed when appropriate to have higher scores reflect less prejudicial attitudes). In the final Logistic regression analyses, each dimension was separately coded, which was equal to 0 (lower stigma) if participants corresponded correctly to all items retaining in their respective dimensions, and is 1 otherwise (higher stigma) [19].

#### Independent variables

As described in the background section (Fig. 1), eleven factors previously shown to be associated with stigmatizing attitudes were taken as independent variables. Consistent with our previous study [36], a 12-item scale of Yes/No/I do not know questions (α = 0.75) was used to measure HIV-related knowledge and also dichotomized into high and low based on its median value (median 10 scores). HIV- related unsafe sexual behaviors was measured by first asking participants whether they had engaged in any form of sexual behavior. Those who answered “yes” were then required to provide information about their unsafe sexual behaviors. In this study, unsafe sex was treated as a dummy variable, which was equal to 1 if respondents had more than two sexual partners (multiple partners) within the past 6 months [36], or had ever had sex with a casual or commercial sex partner [36], or failed to use condoms consistently in every act of sexual intercourse [36], and was 0 otherwise.

The respondents were asked for their age, but they were provided with four possible choices:① younger than 18 years old; ②18–25 years old; ③26–29 years old; ④30 years or older. Furthermore, Chinese university students in the same grade are almost of the same age. Therefore, grade was crudely used to reflect the undergraduates’ age, which was scored on a four-point scale: freshmen, sophomore, junior, and senior. However, in this study, we are particularly interested in whether freshmen have less discriminatory attitudes towards PLWH, since freshmen are required to take an HIV prevention course to curb the HIV epidemic on college campus in the Guideline on HIV Prevention Education for College Students issued by the Ministry of Education and Health of China, according to a report from Beijing Evening News [41]. Therefore, in order to capture its potential effect, grade is transformed from a four-category variable to a single dummy variable, which equals one if the undergraduates were freshmen, and 0 otherwise.

Having ever being tested for HIV, willingness to utilize HTC service, and awareness of the national AIDS policy were respectively assessed through the following three Yes/No questions: 1) Have you even been tested for HIV? 2) If you were offered free HTC service, would you wish to accept? 3) Do you know the Four Frees and One Care policy?

Consistent with our previous study [36], self-perceived risk of HIV infection was measured by asking “what are your chances of catching HIV?” Those who answered “no possibility” were as categorized as “No”, all others (Not sure, Low, Moderate and High possibility) were categorized as “Yes”.

#### Statistical analysis

Raw data collected via the website “www.sojump.com“were first transformed into an Excel file, then double-cleaned and analyzed independently by two authors. Descriptive statistics with cross-tabulation was firstly generated for all questionnaire items. Following the procedures of testing measurement instruments recommended by Dullie and colleagues [42], the data set was randomly and equally divided into two similar subsamples [i.e., a derivation sample (Sample 1) and a validation sample (Sample 2)] to derive and validate the latent variables. Prior to performing the factor analyses, the overall Kaiser-Meyer-Olkin (KMO) statistic and Bartlett’s test for sphericity were calculated to evaluate whether the sample was suitable for performing a factor analysis. The varimax rotation of the principal component analysis [43] was chosen to test the dimensionality of scale items and loading strength of items on factors, to decide on item retention, because none of the variables are normally distributed. The factors with eigenvalues greater than one were retained and an item was assigned to a factor if its factor loading was equal to or higher than 0.40 and did not load on multiple factors [44].

After conducting EFA, maximum likelihood confirmatory factor analysis was then conducted on the validation sample using Amos 24.0 software to verify the factor construct identified in the above factor analyses. An insignificant chi-square statistic (*p* > 0.05) is commonly used to indicate a well-fitting model. However, a large sample size makes it usually impossible to occur [42]. Therefore, three other common goodness-of-fit indicators, including comparative fit index (CFI), goodness-of-fit index (GFI) and the root-mean-square error of approximation (RMSEA), were used to compare the hypothesized structure identified by the EFA with the research data. A model can be considered as a good fit when both CFI and GFI were more than 0.90, and RMSEA was less than 0.08 [42].

Cronbach’s alpha coefficients were also calculated to assess internal consistency reliability of each factor identified in the above factor analyses, and values greater than 0.70 were considered statistically acceptable [43]. Finally, multivariate Logistic regression models using backward LR method were chosen to identify statistically significant variables affecting different dimensions of HIV-related stigma. All *P* values less than 0.05 were taken as statistically significant. Adjusted odds ratio (AOR) and 95% confidence interval (CI) were also calculated. All the data analysis was conducted using IBM SPSS Statistics Version 24.0.

## Results

### Characteristics of participants

**Fig. 2:**
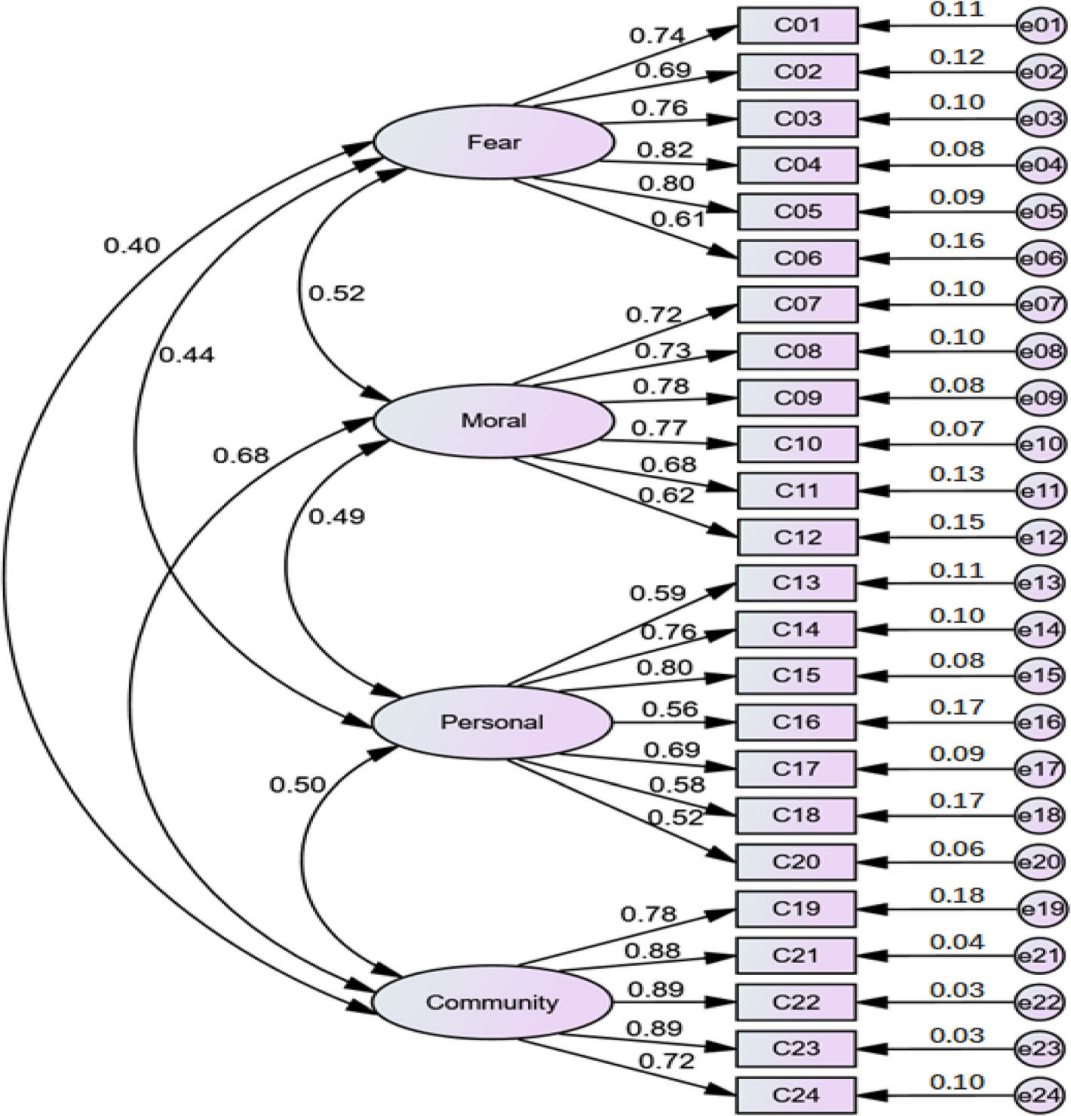
A map displaying the provincial distribution of 10,665 undergraduates was drawn using Supermap iDesktop 8C (2017) and then converted into Microsoft Word format. Excluding Taiwan, Hong Kong, and Macao, there are 31 provinces in mainland China. The exact number in the map indicated that the 10,665 participants were unevenly distributed across 30 provinces (except for Tibet with white highlighted), and were mainly (7200) recruited from Hubei province

A total of 12,750 participants were enrolled in the present study and the effective data utilization rate was 83.6% after excluding 2085 participants without meeting the above-mentioned four criteria. The eligible 10,665 participants were unevenly distributed across 30 provinces in mainland China (except for Tibet) and were primarily (67.5%) recruited from Hubei province (See Fig. 2). Table 1 shows social-demographic characteristics and HIV-related knowledge, attitude, beliefs and behaviors of the 10,665 undergraduates in mainland China of which 57.5% were females, 67.6% were from rural areas, and 29.9% majored in medical science. Nearly three-fourths (71.8%) had already completed more than 1 years of college study (i.e., non-freshmen). Beyond our expectation, 11.5% identified themselves as non- heterosexuals. HIV-related knowledge was lacking with 38.3% of undergraduates answering less than 10 out of the 12 basic questions correctly. Around one-tenth (8.9%) of undergraduates admitted to engaging in unsafe sexual behaviors (including having multiply sexual partners or casual sex, or failing to use condoms consistently) and 48.2% perceived themselves to be at risk of contracting HIV. Although 83.4% of participants expressed the willingness to utilize HTC service, only 7.7% reported to have ever been tested for HIV. In addition, 66.2% were still unaware of the national AIDS policy, which had already been implemented for almost 15 years.

**Table 1:** Social-demographic characteristics and HIV-related knowledge and behaviors of the 10,665 undergraduates in China

Variable	Total sample (*N* = 10,665)		Sample 1 (*n* = 5373)		Sample 2 (*n* = 5292)		χ2	*P*
	n	%	n	%	n	%		
X1: Gender								
0=Female	6137	57.5	3090	57.5	3047	57.6	0.005	0.94
1=Male	4528	42.5	2283	42.5	2245	42.4		
X2: Residential areas								
0=Rural	7207	67.6	3648	67.9	3559	67.3	0.502	0.48
1=Urban	3458	32.4	1725	32.1	1733	32.7		
X3: Major								
0=Non-Medical	7472	70.1	3756	69.9	3716	70.2	0.125	0.72
1=Medical	3193	29.9	1617	30.1	1576	29.8		
X4: Grade								
0=Freshmen	3008	28.2	1536	28.6	1472	27.8	0.784	0.38
1=Non-freshmen	7657	71.8	3837	71.4	3820	72.2		
X5: Sexual orientation								
0=Heterosexuals	9434	88.5	4753	88.5	4681	88.5	0.000	0.99
1=Non-heterosexuals	1231	11.5	620	11.5	611	11.5		
X6: HIV-related knowledge								
0=High	6581	61.7	3300	61.4	3281	62.0	0.381	0.54
1=Low	4084	38.3	2073	38.6	2011	38.0		
X7: Unsafe sexual behaviors								
0=No	9715	91.1	4889	91.0	4826	91.2	0.134	0.71
1=Yes	950	8.9	484	9.0	466	8.8		
X8: Having ever being tested								
0=No	9847	92.3	4975	92.6	4872	92.1	1.054	0.31
1=Yes	818	7.7	398	7.4	420	7.9		
X9: Self-perceived risk of HIV infection								
0=No	5526	51.8	2760	51.4	2766	52.3	0.864	0.353
1=Yes	5139	48.2	2613	48.6	2526	47.7		
X10:Willingness to utilize HTC service								
0=No	1774	16.6	907	16.9	867	16.4	0.476	0.49
1=Yes	8891	83.4	4466	83.1	4425	83.6		
X11: Awareness of the national AIDS policy								
0=No	7062	66.2	3545	66.0	3517	66.5	0.275	0.600
1=Yes	3603	33.8	1828	34.0	1775	33.5		

The final sample size of the derivation sample (sample 1) was 5373 and the confirmatory sample (sample 2) was 5292. Table 1 also shows that there were no statistical difference between sample 1 and sample 2 across all characteristics.

### Exploratory factor analysis (EFA)

The Sample 1 (*n* = 5373) satisfied the requirements for carrying out a factor analysis, since the KMO value (0.94) was greater than 0.70 and Bartlett’s test of sphericity was also highly significant (χ2 = 73,832.02, df = 276, and *p* < 0.001). As presented in Table 2, four factors emerged with an eigenvalue greater than one, explaining 63.26% of the total variance. Except for one item (Item 20) grouped with items from a different factor, all other items were successfully assigned to the given factor as expected from the original model. In addition, all items had an acceptable factor loading (≥0.40) on a single factor. The factor loading of each item, detailed eigenvalue and explained variance of each loaded factor were shown in Table 2.

**Table 2:** Results from exploratory factor analysis after varimax rotation and internal consistency of CVZHSS

Item code	Describe Your Opinion about Each Situation	Factor Loading (n = 5373)				Stigma rate (%)
		Fear	Moral	Personal	Community	
C1^R^	You could become infected with HIV if you are kissing PLWH	**0.79**	0.10	0.15	0.08	56.5
C2^R^	You could become infected with HIV if you are exposed to cough of PLWH	**0.67**	0.24	0.16	0.23	33.8
C3^R^	You could become infected with HIV if you are exposed to the saliva of PLWH	**0.83**	0.10	0.11	0.06	61.2
C4^R^	You could become infected with HIV if you are exposed to the sweat of PLWH	**0.80**	0.19	0.10	0.17	44.3
C5^R^	You could become infected with HIV if you are exposed to the urine of PLWH	**0.81**	0.15	0.11	0.11	53.7
C6^R^	You could become infected with HIV if you are playing with PLWH	**0.57**	0.32	0.24	0.16	43.0
C7^R^	HIV is punishment for bad behavior	0.18	**0.70**	0.12	0.26	29.9
C8^R^	It is women prostitutes who spread HIV	0.18	**0.76**	0.10	0.19	31.8
C9^R^	PLWHA are promiscuous	0.16	**0.78**	0.12	0.23	30.4
C10^R^	Only PLWHA caused by blood transfusion should be cared for and treated	0.17	**0.66**	0.13	0.41	24.1
C11^R^	Youths might be badly influenced by PLWHA and participate in illegal activities	0.19	**0.67**	0.18	0.18	41.1
C12^R^	Only PLWHA who stopped illegal activities should be given care and treatment	0.14	**0.61**	0.20	0.26	39.0
C13	Doctors should treat PLWHA the same as other patients	−0.02	0.15	**0.59**	0.36	22.7
C14	PLWHA should be allowed to work with others	0.15	0.11	**0.81**	0.07	42.5
C15	PLWHA should be allowed to participate in social activities	0.10	0.16	**0.78**	0.19	32.1
C16^R^	PLWHA should be segregated	0.29	0.35	**0.40**	0.26	43.1
C17	PLWHA should be treated the same like other patients	0.03	0.17	**0.64**	0.40	23.4
C18	PLWHA but not yet showing symptoms should be allowed to continue teaching	0.22	0.05	**0.69**	0.00	52.8
C19^R^	PLWHA should be abandoned by his/her family	0.17	0.29	0.13	**0.77**	19.2
C20^c^	I am willing to make friends with PLWH^b^	0.20	0.14	**0.60**	0.03	56.1
C21^R^	PLWHA would be dispelled by his/her family	0.17	0.28	0.15	**0.83**	21.1
C22^R^	PLWHA would be insulted by his/her classmates	0.17	0.26	0.17	**0.84**	21.2
C23^R^	PLWHA would be stigmatized and discriminated	0.16	0.28	0.12	**0.85**	18.2
C24^R^	PLWHA would be abandoned by his partner or spouse	0.13	0.27	0.20	**0.69**	29.3
Eigenvalue		2.48	2.06	1.32	9.32	
% of variance accounted for after rotation		10.34	8.60	5.50	38.82	
Cronbach’s alpha		0.88	0.87	0.83	0.92	
Stigma rate (%)^a^		74.4	61.6	79.0	36.5	

R: reverse-coded items. ^a^ based on the whole sample (*n* = 10,665) ^b^ PLWH: people living with HIV

Bold indicating items that can be explicitly assigned to a single factor (factor loading > 0.40 and did not load on multiple factors)

Items 1–6 loaded on “fear of casual transmission”; Items 7–12 loaded on “Moral judgment”; Items 13–18 and Item 20 loaded on “personal stigma”

Item 19 and Items 21–24 loaded on “perceive community stigma”. ^c^ Except I20, all other items loaded on the given factor from the original scale

### Confirmatory factor analysis (CFA)

**Fig. 3:**
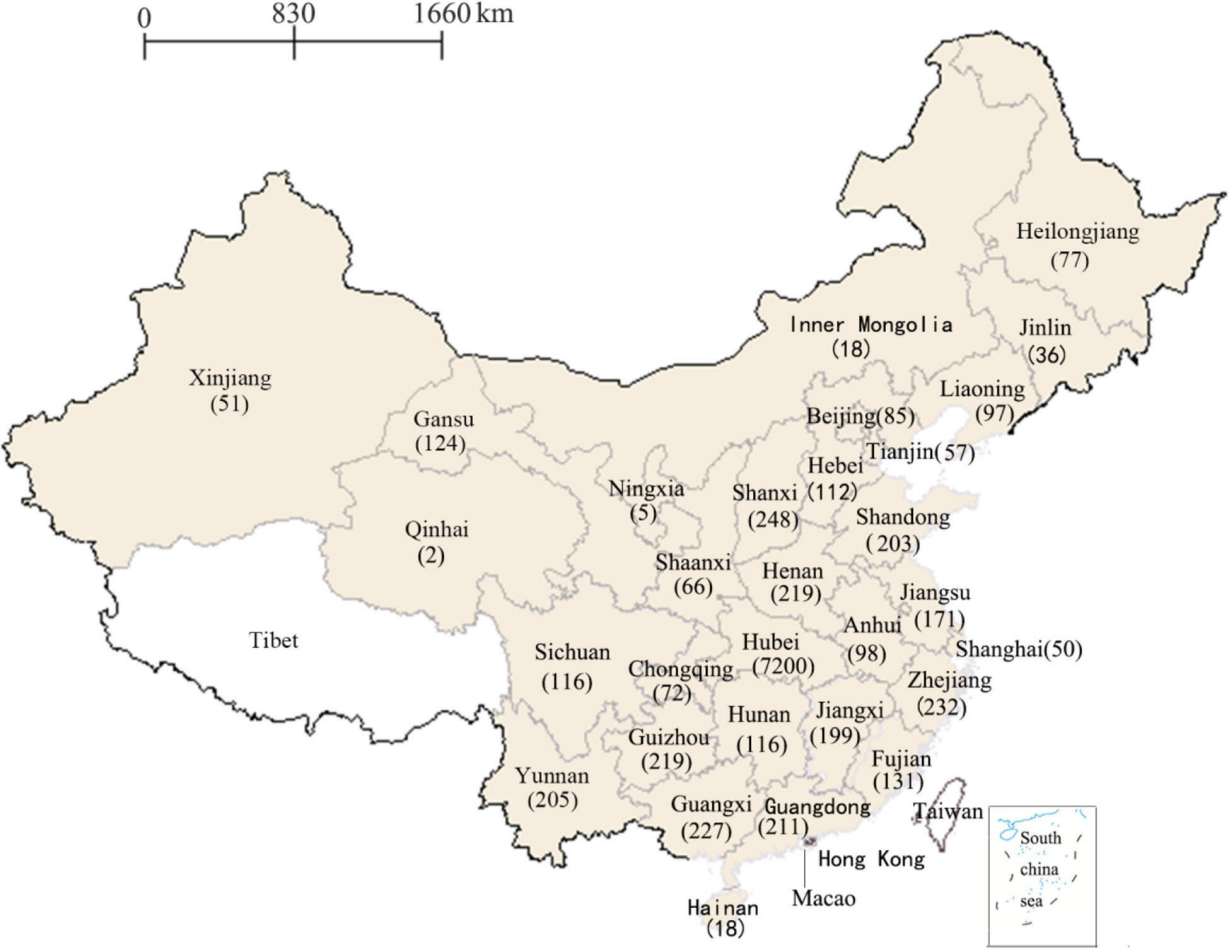
Confirmatory factor analysis: standardized estimates (n = 5292). (χ^2^/df = 24.16, CFI = 0.92, GFI = 0.91, RMSEA = 0.07)

Test of fitness of the above four-factor model on the validation sample (*n* = 5292) yielded a chi-square value of 5942.27, with a degree of freedom of 246 (χ^2^/df = 24.16) and a *P* value less than 0.001, which suggests that the hypothesized model is not entirely adequate. However, as mentioned above, finding a well-fitting model in which a chi-square test is not statistically significant is quite unrealistic, especially in a large sample research. Thus, a significant chi square test (*p* < 0.05) only indicates a need to modify the model for a better fit to the data [40]. Furthermore, based on three other criteria recommended by Dullie and his colleagues [42], the hypothesized four-factor structure was found to have an acceptable fit to the data (Fig. 3), since RMSEA value (0.07) was below 0.08, both CFI (0.92) and GFI (0.91) were above 0.90. In addition, all the structure loading ranged between 0.52 and 0.89 and were significant, indicating a good relationship between the observed variable and latent variable. Although the four domains were positively correlated with each other, they represented different aspects of stigma and could also be independent, providing further evidence that these four domains required separate analyses (See Fig. 3).

### Reliability: internal consistency

As demonstrated in the bottom second row of Table 2, the internal consistency, measured by Cronbach’s alpha, was 0.88 for “fear of casual transmission”, 0.87 for “moral judgment”, 0.83 for “personal stigma”, and 0.92 for “perceived community stigma”, indicating that these four factors had good reliability.

### The level of different dimensions of HIV-related stigma

The percentages of discriminatory responses to each item were displayed in the last column of Table 2. As described above, factor analysis on the 24 items revealed four distinct dimensions, suggesting that each dimension should be considered independently when scoring the CVZHSS. In order to simplify the description, the figures presented here are only the level of four dimensions. As indicated in the last row of Table 2, stigma as measured by fear of casual transmission (74.4%), moral judgment (61.6%), personal stigma (79.0%) and perceived community stigma (36.5%) is highly prevalent. However, it is especially notable that undergraduates indicated a much lower level of perceived community stigma relative to their personal stigma.

### Factors associated with four dimensions of stigma

Separate Logistic regression analyses were performed to identify statistically significant variables affecting four dimensions of stigma. As indicated in Table 3, higher levels of stigma in all four dimensions were consistently associated with non- freshmen, less knowledge about HIV and unsafe sex. Those who had known about the national AIDS policy were less likely to express fear of infection through casual contact (AOR = 0.73, 95% CI = 0.66–0.80), have negative judgment (AOR = 0.77, 95% CI = 0.70–0.83), and indicate personal stigma (AOR = 0.67, 95% CI = 0.60–0.74). Similarly, those who perceived themselves to be at risk of contracting HIV (AOR = 0.81, 95% CI = 0.75–0.88; AOR = 0.80, 95% CI = 0.73–0.88; AOR = 0.76, 95% CI = 0.70–0.83, respectively) and expressed the willingness to HTC service (AOR = 0.74, 95% CI = 0.66–0.83; AOR = 0.56, 95% CI = 0.48–0.66; AOR = 0.70, 95% CI = 0.63–0.78, respectively) had less discriminatory attitudes in three dimensions including moral judgment, personal stigma and perceived community stigma. However, participants from urban areas (AOR = 1.11, 95% CI = 1.02–1.21), non-heterosexuals (AOR = 1.12, 95% CI = 0.99–1.28, *P* = 0.075) and those who had ever been tested for HIV (AOR = 1.33, 95% CI = 1.14–1.54) were more likely to perceive stigma in the community. Relative to students in non-medical degrees, medical students showed lower levels of fear of becoming infected through casual contact (AOR = 0.69, 95% CI = 0.63–0.76) and indicated a lower level of personal stigma (AOR = 0.88, 95% CI = 0.79–0.97). Male students expressed more fear of causal transmission (AOR = 1.10, 95%CI = 1.01–1.21), had more negative moral judgement (AOR = 1.28, 95% CI = 1.18–1.39), and indicated higher levels of perceived community stigma (AOR = 1.54, 95% CI = 1.42–1.68), when compared with their female counterparts.

**Table 3:** Multiple variate Logistic regression analysis of factors associated with different dimensions of HIV-relatefd stigma (*N* = 10,665)

Variables	Fear		Moral		Personal		Community	
	AOR	95%CI	AOR	95%CI	AOR	95%CI	AOR	95%CI
X1: Gender (0 = Female, 1 = Male)	1.10^*^	1.01–1.21	1.28^***^	1.18–1.39			1.54^***^	1.42–1.68
X2: Residential areas (0 = Rural,1 = Urban)							1.11^*^	1.02–1.21
X3: Major (ref: Non-medical)	0.69^***^	0.63–0.76			0.88^*^	0.79–0.97		
X4: Grade (ref: Freshmen)	1.20^***^	1.09–1.33	1.21^***^	1.10–1.32	1.30^***^	1.17–1.44	1.18^***^	1.08–1.30
X5: Sexual orientation (ref: Heterosexuals)							1.12^&^	0.99–1.28
X6: HIV-related knowledge (0 = High, 1 = Low)	2.46^***^	2.22–2.72	1.71^***^	1.57–1.86	2.62^***^	2.34–2.93	2.17^***^	2.00–2.36
X7: Unsafe sexual behaviors (0 = No, 1 = Yes)	1.50^***^	1.26–1.79	1.56^***^	1.34–1.81	1.25^*^	1.04–1.50	2.05^***^	1.78–2.36
X8: Having ever being tested (0 = No, 1 = Yes)							1.33^***^	1.14–1.54
X9: Self-perceived risk of HIV infection (0 = No, 1 = Yes)			0.81^***^	0.75–0.88	0.80^***^	0.73–0.88	0.76^***^	0.70–0.83
X10:Willingness to utilize HTC service (0 = No, 1 = Yes)			0.74^***^	0.66–0.83	0.56^***^	0.48–0.66	0.70^***^	0.63–0.78
X11: Awareness of AIDS policy (0 = No, 1 = Yes)	0.73^***^	0.66–0.80	0.77^***^	0.70–0.83	0.67^***^	0.60–0.74		

^*^P ≤ 0.05, ^**^P ≤ 0.01, and ^***^P ≤ 0.001

^&^ It needs to be pointed that sexual orientation is significant at the 0.10 level, but lost its significance at the 0.05 level (*P* = 0.075)

## Discussion

The primary aim of the study was to validate the CVZHSS among a large undergraduate sample in mainland China. Construct validity was established by conducting both exploratory and confirmatory factor analysis. These two types of results were complementary. This study extends the results by first using the EFA to obtain a modified factor structure for our sample, thus making it possible to make a comparison between the original version and the Chinese version. As expected from the original version, four distinctive dimensions, i.e., fear of casual transmission, moral judgment, personal stigma, and perceived community stigma, were extracted using principal component factor analysis with varimax rotation. One obvious discrepancy is that in our sample, Item20 (“I am willing to make friends with PLWH”) failed to be grouped under the “Perceived community stigma” domain as in the original version. To explain this discrepancy, the wordings of both the original version and the Chinese version were compared. It was found that a translation error was made by changing the subject from “people” (the third person) in the original version to “I” (the first person) in the Chinese version, thus resulting in Item 20 assigned to the “Personal stigma” dimension in our sample.

The following CFA demonstrated that the four-factor model provided a good fit to the data, as evidenced by various model fit indices (CFI = 0.92, GFI = 0.91, RMSEA = 0.07). In addition, the results of reliability analysis also indicated these four factors had acceptable internal consistency (Cronbach’s alpha greater than 0.70). Therefore, the psychometric properties of the Chinese version were found to be largely similar to those of the original version, which showed the construct of HIV- related stigma are consistent across different cultural settings, thereby lending support to a claim that Zelaya’s HIV-related Stigma Scale is a valid, reliable and globally accepted tool [21, 23, 24, 30–34] for comprehensive measure of stigma among HIV- uninfected individuals, and its four distinct domains should be used separately.

The second aim of this study was to identify possible predictors of stigmatizing attitudes among undergraduates in mainland China. The analysis of associations between the four domains of CVZHSS with the other collected variables potentially affecting stigmatizing attitudes among undergraduates has allowed us to confirm data already available in the literature. The findings indicated that except for non-freshmen, less knowledge about HIV and unsafe sex which were consistently associated with higher levels of stigma in all four dimensions, other eight variables including gender, residential area, major, sexual orientation, having ever being tested, perception of HIV risk, willingness to utilize HTC service and awareness of the national AIDS policy played differential roles in affecting different dimensions of stigma.

Consistent with a previous research on the relationship between HIV-related stigma, unsafe sexual behaviors and preventive practices [37], stigmatizing attitude towards PLWH were positively associated with unsafe sexual behaviors (including having multiple sexual partners, causal sex, and failing to use condoms consistently) and were negatively associated with willingness to utilize HTC service. One possible explanation for this finding is that those who had ever engaged in unsafe sex [37] and accepted HIV testing [38] are stigmatized because of their association with the gay community [35, 37] and face strong social pressure. Our data also indicated that non- heterosexuals and those who had ever been tested for HIV were more likely to perceive stigma in their community. Such social environments may lead them to try to justify their stigmatized behaviors by blaming or stigmatizing PLWH [37].

Our study indicated that undergraduates who were knowledgeable about the route of HIV transmission and the national AIDS policy and those who perceived themselves to be at risk of contracting HIV were less likely to have stigmatizing attitudes. This finding is not surprising as it fits the knowledge-attitude-belief-practice model, because the increase of knowledge can dispel misconceptions about HIV transmission via casual contact, increase accurate perception of personal risk, and finally contribute to lower levels of HIV-related stigma.

Another interesting finding is that medical education can significantly reduce fear of casual transmission and personal stigma, but has no significant effect on moral judgments and perceived community stigma. One possible explanation for this phenomenon is that the current dominant biomedical model of health places greater emphasis on providing medical students with the knowledge related to the nature of HIV and AIDS, HIV transmission modes, the risk of professional exposure to HIV, while topics regarding professional ethics, human right, and the relationship between health professionals and patients are often ignored [24].

Consistent with a previous study [28, 45], women were less likely to express stigmatizing attitudes towards PLWH. The increased compassion of females was mainly attributable to social gender roles, especially in China where women are economically, culturally, and socially disadvantaged, and they also shared more responsibility for housework and child-care [28]. Furthermore, gender was also found to exert its indirect effects on stigma through the mediating effect of knowledge, with women having higher knowledge and thus lower levels of stigma than men [45].

Contrary to a previous study [25], this study indicates that respondents from urban areas perceived higher levels of community stigma than their rural counterparts. This is because there existed a more traditional family value (e.g., loyalty, respect, obedience and love) in the rural areas, thus contributing to rural respondents’ lower levels of perceived community stigma. In addition, our study also found that non- freshmen consistently reported higher levels of stigma in all the four dimensions. This could be partially explained by the fact that higher-grade (age) undergraduates, despite having a higher level of knowledge [5], had a higher tendency to report having had sexual debut [5] and were more likely to engage in unsafe sexual behaviors such declining condom use [46], because college student are under less control of their parents and also have more opportunity to interact with opposite gender and same-gender peers [47]. Our data also suggested that the prevalence of unsafe sexual behaviors increased significantly from 6.9% among freshmen to 8.5, 9.4 and 14.1% in senior, junior and senior students, respectively (χ2 = 42.94, *P* < 0.001). Therefore, non-freshmen expressed more negative attitudes toward people living with HIV so as to reduce the resulting discomfort of unsafe sex [37].

### Limitations

Some limitations of this study need to be considered. First, the cross-sectional nature of this study does not allow us to draw causal inferences. Second, questions designed to measure fear of casual transmission (e.g., You could become infected with HIV if you are kissing PLWHA) are hypothetical and may be biased by social desirability. Third, despite of considerable efforts to obtain a large and geographically diverse sample, the extent to which our results can be generalized to undergraduates across the whole country is limited by the fact that we mainly adopted methods of convenience sampling and snowball sampling to distribute questionnaires, thus contributing to the over-representation of undergraduates from Hubei province. Fourth, the questionnaire was anonymously completed by participants, thus resulting in the impossibility of assessing test-retest reliability. Furthermore, criterion validity could not be assessed, because there is currently no “gold standard” scale for measuring of HIV-related stigma in this group. Fifthly, previous study indicated that there were significant differences between stigma perceived by the general negative population and that perceived by HIV-positive individuals [11, 14]. However, HIV- positive students were not excluded from the current analysis, because only a minority (7.7%) of participants reported to have ever been tested for the virus and their HIV infection status was not further assessed with a gold standard diagnostic test. Therefore, it might not be appropriate to directly ask undergraduates whether they themselves held stigmatizing attitude towards PLWH, with the underlying assumption that every participant is HIV-negative. Fortunately, this type of misclassification bias was minimized to a certain extent in this study, because the HIV epidemic in China remains at a fairly low level compared with the global average (e.g., 0.09% vs. 4.6% in 2018), according to the latest figures available from the Chinese Center for Disease Control and Prevention and the Joint United Nations Program on HIV and AIDS. Finally, translation and cross-cultural adaptation of an instrument is a complex and time-consuming process. In order to ensure a valid translation, it is important to invite the instrument’s developers, translators and researchers to deal with potential linguistic, semantic and contextual issues. Due to the limitation of time and resources, this study adapted the Chinese version of Zelaya’s HIV-related Stigma Scale translated mainly by five experts in the filed of psychology, social medicine and nursing in China. Therefore, a translation error identified in the Chinese version contributed to the failure to replicate the original factor structure and loading pattern.

## Conclusions

The lack of a standardized reliable and valid instrument makes it difficult to measure stigma consistently and thus poses a challenge to compare and contrast evaluated interventions. Therefore, there is a need for developing a valid, reliable and globally accepted scale to assess the current level and predictors of HIV-related stigma, and subsequently develop, implement and evaluate anti-stigma interventions. A review of the existing literature indicated that the Chinese version of Zelaya’s HIV-related Stigma Scale (CVZHSS) seems to be a promising measurement tool, because it has more comprehensively assessed stigma than the other three most commonly used scales and has been validated in a relatively large sample of college students and also because it performs well across the wider geographical, political and cultural contexts. However, previous studies have only performed an exploratory factor analysis to test its construct validity among college students with certain demographic characteristic such as currently pursuing a degree in medicine, nursing and education or/and in one city of a certain province (e.g., Hanzhou city of Zhejiang Province). To the best of our knowledge, the current study is the first to evaluate its construct validity in a large undergraduate sample across the whole country combining exploratory and confirmatory factor analytic approaches as well as to examine their respective determinants of four distinct dimensions in this population. Our results indicated the CVZHSS is a reliable and valid measurement tool and can be used to identify undergraduates with high levels of stigma. However, four dimensions of stigma (i.e., Fear, moral judgement, personal stigma and perceived community stigma) were respectively influenced by different determinants and consequently should be treated independently when designing anti-stigma measures.

## Data Availability

The data set supporting the results of this article is available in the Harvard Dataverse repository at: https://dataverse.harvard.edu/dataset.xhtml?persistentId=doi:10.7910/DVN/GBFCTK.

## Abbreviations

AIDS: Acquired Immune Deficiency Syndrome
AOR: Adjusted odds Ratio
CFA: Confirmatory factor analysis
CI: Confidence Interval
EFA: Exploratory factor analysis
HIV: Human Immunodeficiency Virus
HTC: HIV testing and counseling
HUST: Hubei University of Science and Technology
PLWH: People living with HIV
SPSS: Statistical Package for the Social Sciences
